# Exploiting the Prevalence of Homologous Recombination Deficiencies in High-Grade Serous Ovarian Cancer

**DOI:** 10.3390/cancers12051206

**Published:** 2020-05-11

**Authors:** Sara Bouberhan, Lauren Philp, Sarah Hill, Linah F. Al-Alem, Bo Rueda

**Affiliations:** 1 Department of Hematology/Medical Oncology, Massachusetts General Hospital, Boston, MA 02114, USA; sbouberhan@mgh.harvard.edu; 2 Department of Hematology/Medical Oncology, Harvard Medical School, Boston, MA 02115, USA; 3 Division of Gynecologic Oncology, Department of Obstetrics and Gynecology, Massachusetts General Hospital, Boston, MA 02114, USA; lphilp@mgh.harvard.edu; 4 Department of Obstetrics and Gynecology, Vincent Center for Reproductive Biology, Massachusetts General Hospital, Boston, MA 02114, USA; lal-alem@mgh.harvard.edu; 5 Obstetrics, Gynecology and Reproductive Biology, Harvard Medical School, Boston, MA 02115, USA; 6 Department of Pathology, Brigham and Women’s Hospital, Boston, MA 02115, USA; sarah_hill@dfci.harvard.edu; 7 Department of Medical Oncology, Dana-Farber Cancer Institute, Boston, MA 02215, USA

**Keywords:** homologous repair deficiency, BRCA, ovarian cancer, PARP, resistance

## Abstract

High-grade serous ovarian cancer (HGSOC) remains the most lethal gynecologic cancer in the United States. Genomic analysis revealed roughly half of HGSOC display homologous repair deficiencies. An improved understanding of the genomic and somatic mutations that influence DNA repair led to the development of poly(ADP-ribose) polymerase inhibitors for the treatment of ovarian cancer. In this review, we explore the preclinical and clinical studies that led to the development of FDA approved drugs that take advantage of the synthetic lethality concept, the implementation of the early phase trials, the development of companion diagnostics and proposed mechanisms of resistance.

## 1. Introduction

### Incidence, Mortality, Morbidity

Ovarian cancer remains the deadliest gynecologic malignancy, accounting for 13,980 deaths in the United States in 2019 [1]. The symptoms of ovarian cancer are non-specific, and no effective screening test exists [2,3]. The majority of women continue to present with advanced disease (SEER Fact Sheet, Ovary) [4]. Developing strategies to treat advanced disease is critical to the management of ovarian cancer. 

The morbidity related to ovarian cancer is primarily driven by intra-abdominal disease burden, which can cause uncomfortable abdominal distension and frequent bowel obstructions. As such, debulking surgery, or removing all intra-abdominal tumors, is routinely offered to women with advanced disease [2,3]. An “optimal” result of debulking surgeries is to remove all tumor visible to the naked eye, an outcome shown to improve ovarian cancer survival [5]. Women who are not candidates for primary debulking surgery due to extensive disease burden or poor performance status may be offered neoadjuvant chemotherapy followed by an interval debulking surgery [2,3,6].

Following surgical debulking, most women with advanced ovarian cancer are treated with adjuvant platinum-based chemotherapy [7,8,9,10,11,12]. Despite extensive initial surgical resection and combination adjuvant chemotherapy, most women with advanced disease will experience incurable recurrence of their disease within three years, regardless of the adjuvant chemotherapy strategy chosen [7,8,9,10,11,12,13]. Several treatment regimens are available for women with advanced disease [14,15,16]. Response rates and durations of response in the recurrent disease setting are consistently worse than those observed after front line treatment, and median overall survival remains less than five years (SEER Fact Sheet, Ovary). Better therapies for front-line treatment and recurrent disease are urgently needed. Throughout this article we review recent clinical and pre-clinical developments in HGSOC, with a particular focus on the frequent finding of homologous recombination deficiency and its role in the development of new therapies.

## 2. Homologous Recombination Deficiency (HRD)

### 2.1. What Is HRD?

Genomic stability is essential for the accurate transfer of genetic information during mitosis. The cells in our body receive tens of thousands of “hits” to their DNA per day from endogenous or exogenous sources. These negative influences can cause replication stress, which is a major source of genomic instability, often leading to pre-neoplastic lesions and have the potential to contribute to malignant transformation [17]. The constant genomic stress the cell experiences underscores the need for a choreographed DNA damage response (DDR) that performs with high fidelity. Usually, the cell arrests its cell cycle allowing for repair. The type of repair and when it occurs depends on the kind of damage incurred and whether or not the cell is cycling. Six major active cellular pathways repair DNA damage including direct lesion reversal, base excision repair (BER), nucleotide excision repair (NER), mismatch repair (MMR), HR and non-homologous end joining (NHEJ) [18].

### 2.2. BRCA Mutations and HRD

Genetic alterations in the breast cancer 1 (*BRCA*1) and breast cancer 2 (*BRCA*2) pathway comprise the largest number of relevant genomic alterations in high-grade ovarian serous cancers (HGSCs) [19,20]. The relationship between *BRCA* mutations and ovarian cancer is complex and is discussed throughout this review. Both *breast cancer 1* and *breast cancer 2 (BRCA1* and *BRCA2)* genes are prone to mutation, thought to be due to high densities of repetitive DNA elements [21]. As such, mutations in *BRCA1* and *BRCA2* are commonly identified in both hereditary and sporadic cancer [21]. Biallelic loss of *BRCA1* and *BRCA2* is typically a lethal event. However, it was proposed that ovarian and breast tissue-specific factors may permit the persistence of BRCA-null cells, allowing for subsequent tumor formation and loss of the checkpoint p53 function [21]. As such, germline carriers of *BRCA1* and *BRCA2* are predisposed to develop breast and ovarian cancers that have a predictable genomic profile [21,22,23,24].

**Figure 1:**
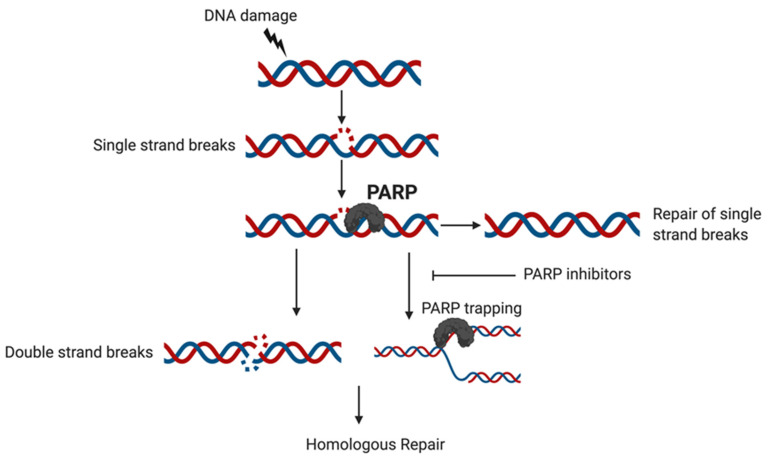
DNA damage may lead to single strand breaks; PARP detects the DNA lesion in the single strand break. PARP takes part in recruiting repair factors to the single stranded DNA lesion site and promotes the activity of enzymes during the repair. PARP inhibitors function by reducing the catalytic activity of PARPs and can help prevent single strand break repair which can lead to double strand breaks which can’t be repaired by BRCA mutant tumors or can trap PARP at the site of DNA damage via preventing PARP detachment from DNA. This then prevents the replication fork from progressing and leads to cell death unless this damage is repaired.

Proteins in the BRCA pathways participate in DDR in two major ways, including the repair of double-strand breaks (DSB) by HR [20] and the protection of stalled replication forks [25]. Essential to the function of these DDR pathways are the PARP enzyme family [26] primarily PARP 1 and 2, which act to repair single-strand DNA breaks (SSB) induced by a variety of endogenous and exogenous sources as shown in Figure 1 [27,28,29].

### 2.3. “BRCAness”: HRD in BRCA Wild Type Tumors

The relationship between germline *BRCA* mutations and the development of ovarian cancer is well established [2,21,22,23,24]. Notably, ovarian cancers related to germline *BRCA* mutations account for only 13% of cases [19]. There are, however, genomic similarities between ovarian tumors arising sporadically and those arising in the setting of germline *BRCA* mutations. Deficient homologous recombination (HR), a hallmark characteristic of *BRCA* deficient tumors, is present in approximately 50% of all ovarian cancers [19]. In the absence of a germline *BRCA* mutation, the finding of deficient HR is often referred to as "BRCAness” [30,31]. Multiple non-germline *BRCA* alterations contributing to HR deficiency have been identified, but each occurs in only a minority of ovarian cancers. For example, homologous recombination relies on a complex of proteins in the Fanconi anemia-BRCA pathway, which is disrupted in approximately 21% of ovarian tumor lines [30,32]. Somatic mutations in *BRCA 1* or *BRCA2* likely occur in 5−10% of tumors [19,30,33]. Additional somatic mutations in genes that play a critical role in DNA repair are another contributor to deficient HR. These alterations include, but are not limited to, RAD51: RAD51 recombinase C, RAD51 recombinase D and BARD1 (RAD51C, RAD51D, and BARD1, respectively), as well as alterations in PTEN, ATM, ATR, and *EMSY* amplification [19,30]. Moreover, frequent *BRCA* promoter methylation events are thought to contribute to this phenomenon [19,31].

### 2.4. HRD and Synthetic Lethality in Ovarian Cancer

**Figure 2:**
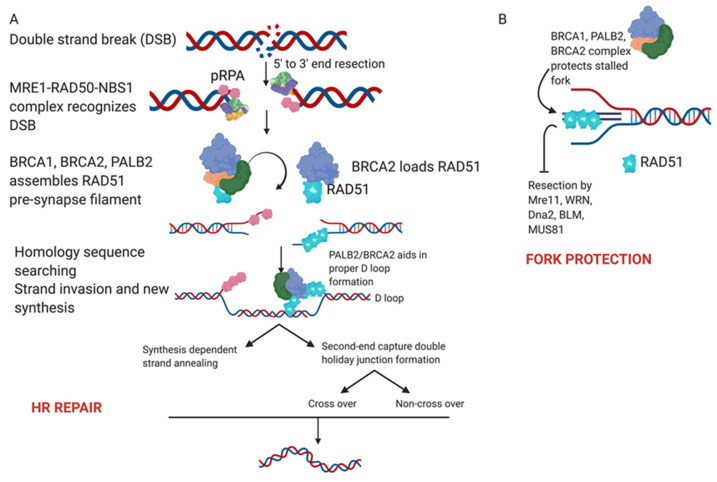
(**A**) Homologous recombination. A DNA double strand break is sensed and recognized by the MRE11-RAD50-NBS1 complex (MRN). The binding of this complex activates ATM kinase that leads to the activation of DNA damage response. Nuclease activity leads to end resection from 5′ to 3′ that then leads to the formation of single-strand DNA ends coated by phosphorylated RPA (pRPA). The exposed single strand DNA activates ATR’s response to facilitate the repair. This leads to the activation of BRCA/BRCA2/PALB2 complex which prepares RAD51 nucleofilaments for loading on DNA. The RAD51 nucleoprotein filament is loaded by BRCA2/PALB2 on the homology sequence and the RAD51 coated strand along with BRCA2/PALB2 mediates strand invasion and D loop formation. This also releases the MRE11-RAD50-NBS1 complex and the double stranded breaks are restored by branch migration, DNA synthesis and ligation. (**B**) Stalled replication forks are protected by the BRCA1/BRCA2/PALB2 complex with RAD51 loading onto the nascent DNA. This protects from end resection by MRE11 and other proteins to allow fork restart.

As mentioned previously, functional DNA repair mechanisms are essential to the integrity and stability of the genome and to preventing tumorigenesis. Cells respond to DNA damage through multiple DNA repair mechanisms or DDR pathways, which exist to sense lesions in DNA, activate response pathways and ultimately repair DNA lesions [34] as shown in Figure 2. Without functional DDR pathways, cancer cells become differentially more sensitive to DNA damaging agents than healthy cells. Platinum-based chemotherapy, the most active cytotoxic chemotherapy for the treatment of epithelial ovarian cancer, works as a DNA damaging agent. Cisplatin and carboplatin form adducts with DNA [35,36] which lead to the development of double-strand DNA breaks [37]. Tumors with homologous recombination defects, including mutations in *BRCA*, are highly sensitive to the DNA damage caused by platinum-based chemotherapy [38,39].

PARP inhibitors exploit defects in DNA damage repair by interfering with additional DNA damage repair pathways, rather than by directly damaging DNA [40]. This "second-hit" to DNA damage repair pathways is theoretically lethal to cancer cells and non-lethal to other somatic cells, distinguishing it from traditional chemotherapy [40,41,42]. This principle, referred to as synthetic lethality [40,43] was the basis for the clinical development of PARP inhibitors in tumors with HRD [40]. The following sections will review the development of PARP inhibitors for the treatment of ovarian cancer and the clinical data available for the five best-studied PARP inhibitors: olaparib, rucaparib, niraparib, veliparib, and talazoparib.

## 3. PARP Inhibitors 

### 3.1. What Is the Importance of PARP? 

As described above the PARP enzyme family [26], primarily PARP 1 and 2, is essential to the repair of single-strand DNA breaks (SSB) which can be induced by a variety of endogenous and exogenous sources [27,28,29]. The PARP enzymes sense sites of DNA damage and bind to these areas through their n-terminal Zn binding domains, inducing a conformational change in the enzyme and activating the catalytic site [26,44]. The PARP enzymes then catalyze the polymerization of ADP-ribose units to target proteins at lysine, aspartic acid and glutamic acid residues to form Poly(ADP) ribose (PAR) chains [45,46]. Target protein PARylation then mediates the recruitment and binding of DNA repair proteins to the damaged site, including X-ray repair cross-complementing 1 (XRCC1), Polynucleotide kinase-phosphatase enzyme (PNKP), Aprataxin and PNKP like factor (APLF) and Aprataxin (APTX). Chromatin remodelers such as amplified in liver cancer protein 1 (ALC1) and Nucleosome remodeling deacetylase (NuRD) and histones, such as H1, are also recruited which together act to complete the process of DNA repair leading to cell survival [26,40,46,47]. However, in cases of severe DNA damage, PARP enzyme activation can alternatively promote cell death through cellular NAD+ depletion and the induction of apoptosis [48]. Once DNA repair is complete, PARP enzymes undergo auto-PARylation which inhibits further DNA-binding and causes PARP to be released from bound chromatin [40,47,49]. The subsequent activation of the cellular PARG (Poly (ADP) ribose glycohydrolase) enzyme then degrades the PAR chains bound to the site of newly repaired DNA [49].

Given the importance of PARP in the DNA-repair pathway, early research was focused on the development of PARP inhibitors, starting with the discovery that small molecule nicotinamine agents could inhibit DNA-PARylation and subsequently increase cell sensitivity to extrinsic DNA damage [50]. Further research led to the development of current day PARP inhibitors, the first being olaparib, a multi-PARP inhibitor, which was FDA approved for the treatment of ovarian cancer in 2014. These drugs are noted to have two main mechanisms of action, the first being disruption of NAD+ binding to the PARP catalytic domain after activation, and the second being prevention of auto-PARylation and subsequent “trapping” of PARP on DNA [51,52,53]. When tested in BRCA-deficient cell lines and xenograft models, PARP inhibition with these novel agents led to highly effective tumor-specific cell killing [54,55]. While the exact mechanisms underlying this effect are still debated, it is thought that PARP activity is required to stabilize DNA replication forks recruited to repair single-strand DNA breaks [56,57]. When PARP is inhibited, these replication forks collapse leading to double-strand DNA breaks, which are unable to be repaired in cells with homologous recombination deficiency such as those with *BRCA* mutations [55,57]. This interaction is known as synthetic lethality, in which a combination of inherited or induced deficiencies in two or more genes or pathways leads to cell death whereas a deficiency in either single gene or pathway does not [58]. More recent evidence suggests that PARP-trapping may be an additional cause of cell lethality after PARP inhibitor treatment [52,56,59]. However, regardless of the mechanism, this synthetically lethal combination of BRCA deficiency and PARP-inhibition was the driver behind the initial clinical trials showing activity of PARP-inhibitors as single-agent treatments in women with germline *BRCA* mutations. 

Currently, three PARP inhibitors are FDA approved for use in patients with ovarian cancer –olaparib (AstraZeneca), rucaparib (Clovis), and niraparib (GlaxoSmithKline) (Table 1). These medications are generally similar with respect to efficacy but do have important differences in toxicity and metabolism related their underlying chemical structures [60,61]. All three approved PARP inhibitors bind PARP-1 and PARP-2 with comparable efficacy; however, rucaparib additionally binds PARP-3 [60,62,63]. Differences in PARP-trapping potency are also noted between drugs, with niraparib having the highest PARP-trapping ability of the approved inhibitors [53]. Additionally, differences are seen between the drugs in terms of pharmacokinetics and distribution, such as niraparib having CNS penetration, even though they are all orally bioavailable [64]. Despite the lack of head-to-head comparison trials it is thought that these differences may only impact dosing and side-effect profile as increased PARP-trapping is associated with worse myelosuppression [63,65].

#### 3.1.1. Olaparib 

Olaparib first received FDA approval as a treatment for patients with germline *BRCA* mutations with ≥ 3 prior lines of treatment based on the results of a number of phase 2 trials [53,62,66,67]. In a study by Kaufman et al. of 193 patients with advanced or recurrent germline-*BRCA* mutated ovarian cancer, olaparib monotherapy resulted in a 31.1% overall response rate in this heavily pre-treated group. Olaparib was subsequently studied as a maintenance therapy, following treatment for platinum-sensitive recurrent disease. Study 19, a phase 2 trial published in 2012 by Ledermann et al, looked at the role of olaparib maintenance therapy in patients with platinum-sensitive recurrent ovarian cancer treated with ≥ 2 prior lines of chemotherapy [68]. Of 265 patients enrolled, 136 received olaparib maintenance and had a significant improvement in progression free survival (PFS) from 4.8 months in the untreated group to 8.4 months in the olaparib treated group (HR 0.35, *p* < 0.001). However, no difference was noted in overall survival. Only 22.8% of olaparib patients had a known germline *BRCA* mutation, and a subgroup analysis showed the largest benefit in this group, although benefit was also seen in the *BRCA*-unknown patients [68]. A larger overall survival benefit was noted in patients with a *BRCA* mutation at 4.7 months, and the most marked survival advantage in all groups was noted after 36 months, highlighting the potential for long-term survival with PARPi treatment. In fact, almost 25% of patients continued on treatment after 2 years and 11% received maintenance for over 6 years. These results were confirmed in the phase 3 SOLO-2/ENGOT OV-21 study which randomized patients with recurrent germline-*BRCA* mutated platinum sensitive ovarian cancer after ≥ 2 prior lines of chemotherapy to olaparib monotherapy maintenance versus placebo [70]. A remarkable improvement in PFS was noted in the olaparib maintenance arm at 19.1 months vs. 5.5 months in the placebo arm (HR 0.3 (0.22–0.41), *p* < 0.0001). No difference in health-related quality of life was noted in the olaparib maintenance arm as compared to placebo, leading to the conclusion that olaparib is well tolerated and effective in the maintenance setting [76]. FDA approval was granted in 2017 for olaparib use as maintenance therapy after response to platinum-based chemotherapy in patients with biomarker-independent recurrent ovarian cancer [53,62].

In 2018 olaparib was granted its most recent approval for maintenance after first-line chemotherapy in patients with germline or somatic *BRCA* mutations based on the results of the phase 3 SOLO-1 trial [53,62,71]. This study enrolled 391 patients with newly diagnosed stage 3 or 4 ovarian cancer treated with either neoadjuvant or adjuvant platinum-based chemotherapy who were known to have either a germline or somatic *BRCA* mutation. Patients were randomized 2:1 to olaparib maintenance or placebo for two years. At three years of follow-up, 60% of patients in the olaparib group were alive and recurrence free versus only 27% of patients in the placebo arm (HR 0.30 (0.23–0.41), *p* < 0.001) corresponding to a 36-month improvement in overall PFS. Furthermore, this advantage appeared to continue past 24 months, suggesting that there may be a sustained benefit to PARPi therapy beyond the completion of treatment [71].

The recently published PAOLA-1/ENGOT-Ov25 trial (2019) investigated the combination of olaparib plus bevacizumab maintenance in women with newly diagnosed advanced ovarian cancer [77]. PAOLA-1 was the first phase 3 study investigating the combination of a PARP inhibitor with bevacizumab in the maintenance setting [77]. The combination of bevacizumab, an anti-angiogenic VEGF-inhibitor, plus platinum-based chemotherapy followed by bevacizumab maintenance had been previously shown to improve PFS in this population [10,11]. Of the 806 patients randomized, only 30% had known *BRCA* mutations while 48% of patients had HRD, defined by a *BRCA* mutation or a score of ≥ 42 on the Myriad myChoice HRD test [77]. The combination of bevacizumab and olaparib improved PFS in the overall cohort by 5.5 months (HR 0.59 (0.49–0.72), *p* < 0.001) and by 19.5 months in patients with HRD-positive tumors (HR 0.33 (0.25–0.45)). The combination treatment also improved PFS in patients without a *BRCA* mutation; however, to a lesser degree (HR 0.71 (0.58–0.88), median PFS improvement of 2.9 months). However, given the lack of an olaparib only arm in the trial, it is unclear if the benefit in the non-*BRCA* mutated patients is due to the effect of olaparib alone or due to a synergistic effect of olaparib and bevacizumab. Furthermore, given the increased toxicity seen in the combination arm, the role of this combination in clinical practice is still to be determined.A number of other phase 3 trials are currently underway, looking at the use of olaparib in novel combinations with other agents (Table 2). 

#### 3.1.2. Rucaparib

The ARIEL 2 Part 1 study was a phase 2 study of daily rucaparib treatment in 206 patients with platinum sensitive recurrent ovarian cancer who had received ≥ 1 previous treatment regimen [72]. Patients were further stratified into three pre-defined HR subgroups. Patients were defined as *BRCA* mutants (germline or somatic), *BRCA*-wild type with loss of heterozygosity (LOH) high or *BRCA*-wild type with LOH low. Median progression-free survival on rucaparib treatment was noted to be significantly longer in the *BRCA*-mutant group (12.8 months, HR 0.27 (0.16–0.44)) and LOH-high group (5.7 months, HR 0.62 (0.42–0.90)) compared to the LOH-low group (5.2 months). This study demonstrated that deficient HR status (HRD) may confer a benefit during PARPi treatment regardless of *BRCA* mutation status. The results of ARIEL-2 led to the first FDA approved indication, in 2016, for rucaparib as treatment in recurrent or progressive platinum-sensitive ovarian cancer with either somatic or germline *BRCA* mutations after ≥ 2 prior lines of chemotherapy [53,62].

To investigate the use of rucaparib in the maintenance setting, a large prospective phase 3 trial (ARIEL-3) recruited 564 patients with recurrent platinum-sensitive ovarian cancer who had either a complete or partial response to their last chemotherapy cycle [73]. Patients were randomized 2:1 to rucaparib maintenance at 600mg PO BID or placebo. Across the cohort, 35% of patients had a *BRCA* mutation and 28% were *BRCA*-wild type but LOH high. Median progression-free survival was noted to be significantly longer overall in the rucaparib group as compared to the placebo group (10.8 vs. 5.4 months, HR 0.36 (0.30–0.45)) with even more impressive progression-free survival advantages noted in the HRD group (13.6 months vs. 5.4 months, HR 0.32 (0.24–0.42)) and *BRCA*-mutant group (16.6 months vs. 5.4 months, HR 0.23 (0.16–0.34)). The rucaparib group was noted to have a significantly higher rate of grade 3+ adverse events at 56% vs. only 15% in the placebo group, the most common being anemia and elevated liver enzymes. In 2018, the results from ARIEL-3 lead to the second FDA approved indication for rucaparib as a maintenance therapy following treatment of platinum-sensitive recurrent ovarian cancer, regardless of *BRCA* status [53,62]. Three large phase 3 trials are currently planned to investigate the use of rucaparib as primary treatment of *BRCA*-mutated ovarian cancer and in combination with other agents for novel maintenance strategies (Table 3). 

#### 3.1.3. Niraparib

The results from the phase 3 NOVA study resulted in FDA approval in 2017 for niraparib as maintenance therapy for recurrent ovarian cancer with a complete or partial response to previous platinum-based chemotherapy. The Myriad myChoice HRD assay was used to identify HRD-positive tumors in the non-*BRCA* subgroup. The NOVA trial again demonstrated that in a cohort of biomarker unselected patients with platinum-sensitive recurrent disease, niraparib maintenance improved PFS over placebo treatment in all three efficacy groups (germline *BRCA* mutated (HR 0.27 (0.17–0.41)), non-*BRCA* HRD positive (HR 0.38 (0.24–0.59)) and *BRCA* wildtype (HR 0.45 (0.34–0.61)) [74]. Patients with *BRCA* germline mutations had a median PFS of 21.0 months. Patients in the non-germline *BRCA*, HRD positive cohort had a PFS of 12.9 months, and patients in the overall non-germline *BRCA* cohort had a PFS of 9.3 months. 

Niraparib was also approved in 2019 for the treatment of patients with advanced or recurrent HRD-positive disease after ≥ 3 prior therapies [53,62,74]. A large, single-arm phase 2 trial, the QUADRA trial, investigated the utility of niraparib monotherapy for late-line treatment of recurrent ovarian cancer [75]. Of 463 patients enrolled, 33% were considered platinum resistant and 35% were platinum refractory; 48% had HRD positive tumors, including *BRCA*-mutations. In the cohort of patients with measurable disease at baseline, 10% of patients had an overall response to niraparib treatment. When only platinum-sensitive, PARP-naive patients with HRD tumors were examined, the response rate was higher at 28%, with 68% of the cohort achieving at least disease control. Responses in both groups were durable with a median duration of response between 9.2–9.4 months. 

Niraparib combinations have also been studied in phase 2 trials including the TOPACIO / Keynote-162 study of niraparib plus pembrolizumab as treatment for advanced platinum-resistant ovarian cancer [78] and subsequently in the AVANOVA2/ENGOT-ov24 study of niraparib plus bevacizumab versus niraparib alone in platinum-sensitive recurrent ovarian cancer [79]. In the TOPACIO study, investigators noted an overall response rate of 18% and a disease control rate of 65%, which included 3 complete responses. In the patients with a complete or partial response, the median duration of response had not been reached at 12.4 months of follow up, and the response rate appeared independent of HRD or PD/PD-L1 status. In the AVANOVA2/ENGOT-Ov24 study, 97 patients were enrolled and randomized 1:1 to niraparib or niraparib plus bevacizumab until disease progression. The majority of patients had received 1-2 prior lines of treatment and overall approximately 60% of patients had HRD-positive tumors based on the Myriad myChoice HRD test. Niraparib plus bevacizumab resulted in significantly longer PFS than niraparib alone in the entire cohort (HR 0.35 (0.21–0.57)), in HRD positive patients (HR 0.38 (0.20–0.72)) and in HRD-negative patients (HR 0.40 (0.19–0.85) at a median follow up of 16.9 months. 

Most recently, the PRIMA/ENGOT-Ov26 study looked at the use of niraparib as maintenance therapy in patients with newly diagnosed advanced ovarian cancer and found that niraparib maintenance significantly improved PFS after response to platinum-based chemotherapy when compared to placebo (HR 0.62 (0.50–0.76)) [80]. However, the improvement in PFS in the overall cohort was small at only 5.6 months when compared to the improvement seen in the HRD-positive cohort at 11.5 months. Correspondingly, the largest PFS benefit was noted in the HRD-positive group with a HR of 0.43 (0.31–0.59) for progression. Interim survival data did not show a significant improvement in overall survival in the niraparib arm but did suggest a trend in this direction (HR 0.70 (0.44–1.11). Similar to other PARPi, a number of phase 3 trials are underway to investigate novel indications and combinations for niraparib in ovarian cancer (Table 4). 

#### 3.1.4. PARP-inhibitors under investigation for use in Ovarian Cancera.

(a) Veliparib

Pre-clinical and phase 1 studies of veliparib have demonstrated activity against both newly diagnosed as well as recurrent platinum-resistant and platinum-sensitive ovarian cancer [81,82,83,84]. A phase 2 trial published by Coleman et al. in 2015 also reported a moderate response rate of 26% to veliparib monotherapy at a dose of 400 mg BID in the treatment of persistent or recurrent *BRCA*-mutated cancers [85]. Furthermore, studies of veliparib combinations with chemotherapy and bevacizumab have also reported reasonable response rates and the ability to combine veliparib with standard chemotherapy doses without excessive toxicity [81,82,83]. Based on these results, a large phase 3 trial investigated the use of veliparib given concurrently with standard-dose platinum-based chemotherapy followed by maintenance veliparib (“veliparib throughout treatment”) in newly diagnosed advanced ovarian cancer [86]. The VELIA trial demonstrated a PFS benefit to veliparib throughout treatment in the overall cohort (HR 0.68 (0.56–0.83), median PFS 23.5 months), however the benefit was most pronounced in patients with BRCA mutations (HR 0.44 (0.28–0.88), median PFS 34.7 months) followed by patients with HRD-positive tumors (HR 0.57 (0.43–0.76), median PFS 31.9 months). Furthermore, the number of planned chemotherapy doses and median number of chemotherapy cycles were similar across all arms although there was a higher rate of grade 3 and 4 adverse events in the veliparib throughout group. Despite being the first PARPi phase-3 study to demonstrate efficacy when combined with first-line chemotherapy, these findings have not yet resulted in an FDA approval for veliparib. 

(b) Talazoparib 

Talazoparib is a novel PARP inhibitor developed by Pfizer Inc. which received FDA approval in 2018 for use in *BRCA*-mutated HER-2 negative locally advanced or metastatic breast cancer [87]. When compared to olaparib and rucaparib, talazoparib inhibits PARP enzymatic activity at a similar rate but traps PARP-DNA complexes up to 100 times more effectively suggesting it may be the most potent PARPi developed to date [52,88]. Phase 1 basket trials have demonstrated efficacy in advanced *BRCA*-mutated ovarian cancers [89,90], however, at this point in time there is limited phase 2 or 3 evidence suggesting a role for talazoparib in the treatment of ovarian cancer. 

### 3.2. Expanding the Use of PARP Inhibition with HRD Testing 

All women with ovarian cancer are recommended to undergo germline genetic testing for *BRCA1* and *BRCA2* (Society for Gynecology Clinical Practice Statement 2014 [91]). This recommendation was intended to identify *BRCA* carriers to appropriately test and counsel at risk relatives. A second outcome of this recommendation is that the identification of a germline *BRCA* mutation confers a very high likelihood her tumor exhibits HRD, as previously discussed. In effect, germline *BRCA* testing was the first test for HRD. The success of PARP inhibitor therapy for the treatment of *BRCA* mutated ovarian cancer prompted considerable research to develop a clinical assay to detect HRD in non-*BRCA* mutated tumors. The following section reviews the currently available clinical assays as well as ongoing research to identify better methods to detect HRD. 

As previously discussed, approximately 50% of ovarian cancer tumors have defects in the HR pathway [19,92]. A frequent cause of HRD in the absence of a germline *BRCA* mutation is somatic mutation of *BRCA1* or *BRCA2* [19]. Testing for somatic *BRCA* mutations is frequently used in clinical practice, and is supported by multiple commercial platforms. Several caveats, however, should be considered when interpreting somatic *BRCA* results. Somatic testing uses a heterogeneous sample, which contains cancer cells intermixed with normal cells. The specimen needs to be of high-quality sampling to reach a reliable result. Somatic mutations may be different between tumors from different sites. Lastly, somatic mutations may not be stable with time [93]. The importance of identifying non-BRCA mutations in patients to be treated with PARP inhibitors became quickly evident. Davies et al. has developed a weighed model (HRDetect) that is able to distinguish six different signatures that predict BRCA deficiency. They aimed to develop this model based on previous studies showing that a mutation in genes such as *BRCA1/2* leads to at least five different mutational signatures [94,95]. They utilized this information to define genomic features of BRCA1/2 deficiency to develop a whole genome sequencing-based trained predictor to detect HR-deficient tumors. Their dataset was collected from 560 patients where somatic base substitutions, small indels and rearrangements were detected. Using HRDetect, they were able to identify molecular features if BRCA was inactivated through the germline mutation, somatic mutation, hypermethylation or other methods of inactivation. They show that they can determine mutational signatures that were able to identify a larger proportion of breast cancer patients (~22%) than previously identified (~1–5%) [96]. The importance of genomic analysis and identifying target DNA repair mutations and variants has been extensively investigated in ovarian and breast cancer [97,98], which paved the path for multiple companies to design companion diagnostic platforms for clinical use.

There are several companion diagnostic platforms that have emerged and are FDA approved, marking milestones in precision therapies. FoundationOne CDx from Foundation Medicine was approved in 2017. This is a next generation sequencing platform detecting substitutions, insertions and deletion alterations, and copy number alterations in 324 genes and gene rearrangements, as well as genomic signatures including microsatellite instability (MSI) [99] and tumor mutational burden (TMB). A list of alterations reported by Foundation OneCDx can be found in Supplemental Tables S1 and S2. Foundation OneCDx now includes a loss of heterozygosity (LOH) score for all patients with ovarian cancer. By measuring genome-wide loss of heterozygosity, a measure of genomic scarring, the FoundationOne Cdx LOH score can be used to predict HRD. This companion diagnostic was used in the ARIEL3 trial, and identified a subgroup of *BRCA*wt patients who had greater benefit from PARP inhibitor maintenance [73]. 

Another companion diagnostic is Myriad myChoice HRD, which was approved in 2019 and is currently the most comprehensive HRD test. Myriad previously had gained approval for the BRACAnalysis in 2014 for the use of olaparib and rucaparib in ovarian cancer. In contrast to the BRACAnalysis companion diagnostic, myChoice measures single nucleotide variants, insertions and deletions, and large rearrangement variants in protein coding regions and intron/exon boundaries of the *BRCA1* and *BRCA2* genes and determines a genomic instability score (GIS) [93,100]. The patient’s HRD score is generated based on three measures: Loss of Heterozygosity (LOH) [101], Telomeric Allelic Imbalance (TAI) [102], and Large-scale State Transitions (LST) scores [93,103,104]. These scores are then used to aid in determining which ovarian cancer patients are likely to benefit from PARP inhibitor treatments, where an HRD score ≥ 42 is considered positive. MyChoice is currently marketed as a companion diagnostic for niraparib in ovarian cancer. The new indication for use of niraparib in ovarian cancer was primarily based on the phase 2 QUADRA trial NCT02354586 The trial tested the use of niraparib in women who received three or more treatments for advanced ovarian cancer, and showed that women with HRD+ scores had more benefit from niraparib [75]. The FDA approval of niraparib in ovarian cancer was concurrent with the myChoiceHRD companion diagnostic approval. Ovarian cancer patients whose tumors have a positive GIS Status and/or pathogenic variants in *BRCA1* or *BRCA2* are considered good candidates for treatment. myChoice HRD identified about twice as many patients for treatment with niraparib compared to their previous BRACAnalysis CDx test. It is worthy of note that the myChoice companion diagnostic is only approved for use in ovarian cancer. 

Recently, a computational tool called signature multivariate analysis (SigMA) was developed by Gulhan et al. [105]. Their preliminary data shows that SigMA can be used to accurately detect the mutational signatures associated with homologous repair deficiencies from targeted gene panels. Sigma utilizes a simulated prediction likelihood approach which can recapitulate whole genome sequencing results using panels typically used in hospitals. This algorithm is able to identify mechanisms that underly mutations using existing whole genome sequencing data to determining co-occurring signatures that may further stear treatment strategies. Their data is supported by drug response data to olaparib, rucaparib and others. The authors’ data suggests that the signatures they are measuring can collect information even from low mutational counts, which could potentially identify ovarian cancer patients who would benefit from PARP inhibitors and would have been missed using the traditional techniques [105]. Although not FDA approved yet, this technology sheds some light on the benefits of harnessing the use of artificial intelligence and computational biology in patient stratification for PARP inhibitor treatment. 

### 3.3. Replication Combing Assay (RCA)

**Figure 3:**
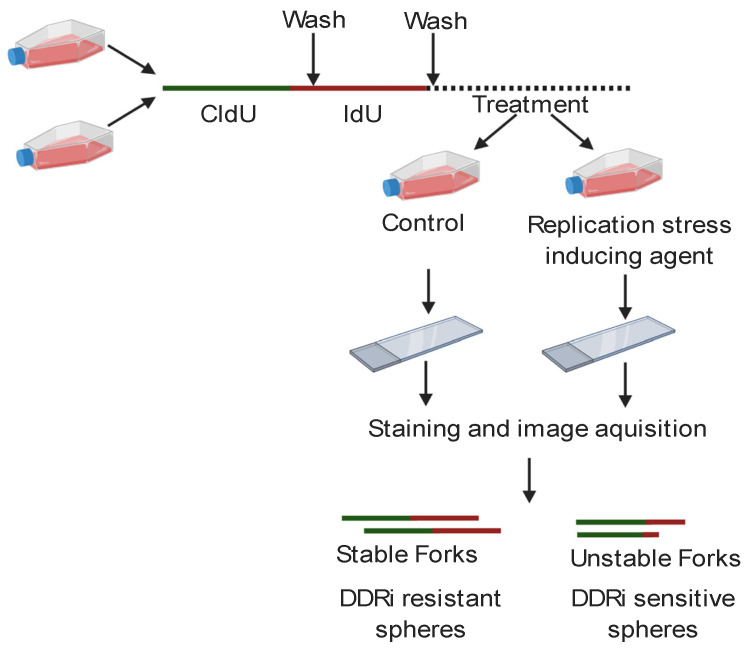
Schematic of DNA fiber technique. Cells are sequentially pulsed with the nucleotide analogs CldU and IdU and then treated with a replication stress inducing agent such as hydroxyurea. Cells are lysed, the DNA fibers are linearly arrayed on cover slips and stained with fluorescently tagged antibodies, and the fibers are visualized and counted. The ratio of the green first track to the red second track is calculated for each fiber. Tumor cells with stable forks that are more likely resistant to DDR inhibitors (DDRi) have a ratio of one while tumor cells with unstable forks have a ratio less than one and are more likely sensitive to DDRi.

In addition to strategies to detect genomic scarring, a novel approach called a replication combing assay (RCA) may offer a dynamic assessment of BRCA family protein function. Although HR and stalled fork protection share some BRCA family protein partners, they are ultimately thought to be separate functions [106,107,108,109]. Thus, different clinical assays will be needed to decipher which DDR defects are present in each unique HGSC. One possible method of assessing for stalled replication fork defects could be to perform RCA on organoid models derived from each patient’s tumor [110]. Organoids are three-dimensional models derived from patient samples, which both morphologically and molecularly mimic the parent tumor from which they were derived. Preliminary studies indicate that stalled fork protection defects detected by the RCA performed on patient-derived organoids may correlate with sensitivity to carboplatin, gemcitabine, and CHK1 and ATR inhibitors [110]; however, a larger number of patients must be assessed to determine the predictive value of this assay. In short, for the RCA, patient-derived organoids are pulsed sequentially with two different nucleoside analogs, followed by treatment with the replication fork stalling agent hydroxyurea [111] as shown in Figure 3. 

The organoids are lysed and the replicated DNA fibers which incorporated the nucleoside analogs are linearly arrayed on special coverslips and each analog is stained with different antibodies such that the analog incorporated first is fluorescently labeled green and the analog incorporated second is labeled red [110]. The fibers are scored for the ratio of second to the first track (e.g., red to green). If the tracks are the same length and the average ratio is one, then the tumor was able to protect its replication forks and should be resistant to the above agents. If the second track is degraded and the average ratio is less than one, the tumor has a replication fork protection defect and may be sensitive to the above agents [110]. Though cumbersome, with proper clinical validation, this assay may provide predictive value for a large array of therapeutic agents in HGSOC.

## 4. PARP Inhibitor Resistance

Almost all patients who initially demonstrate response to PARP inhibition will experience disease progression following prolonged PARP inhibitor exposure [70,71,73,74,77,80,86] PARP inhibitor resistance is likely multifactorial [112,113] and multiple contributing mechanisms have been proposed. 

### 4.1. Mechanisms of PARP Inhibitor Resistance

Conceptually, PARP inhibitor resistance can be divided into mechanisms that restore HR and mechanisms that are independent of HR. Restoration of HR by reversion mutations in *BRCA1* or *BRCA2,* for example, is a well described mechanism of PARP inhibitor resistance [112,113]. Somatic reversion mutations and intragenic deletions restoring function of BRCA1 and BRCA2 in patients with known germline *BRCA* mutations have been well described in chemotherapy-resistant ovarian cancer [114,115]. *BRCA* reversion mutations have been identified in tissue specimens and blood-based cell free DNA. A recent study using a cell-free DNA assay identified *BRCA* reversion mutations in approximately 18% of women with platinum-resistant disease [116]. Presence of a reversion mutation was associated with resistance to rucaparib [116], as expected, but the findings of this study suggest that reversion mutations likely account for only a minority of cases of PARP inhibitor resistance. Notably, the frequency of reversion mutations following prolonged PARP inhibitor treatment has never been systematically studied in a prospective fashion, and the clinical relevance of an incidentally identified *BRCA* reversion mutation has not been clarified. 

*BRCA* reversion mutations are only one of several resistance mechanisms that restore HR. Under the pressure of PARP inhibition, the ability of a hypomorphic BRCA1 protein to be stabilized by heat shock protein 90 (HSP-90) has been described [117]. The newly-stabilized BRCA1 protein was able to interact with the PALB2-BRCA2-RAD51 complex, strongly suggesting restoration of HR. This alteration conferred resistance to both cisplatin and PARP inhibition in vitro [117]. Additionally, alternative splicing variants of *BRCA1* have been shown to restore the *BRCA1* reading frame, conferring platinum and PARP inhibitor resistance in vitro [118]. While intriguing, the frequency of these alterations in clinical practice remains unknown.

Multiple other mechanisms of resistance in the absence of proficient HR have been proposed [113]. The ovarian cancer stem cell population, which has been implicated in resistance to chemotherapy [119,120,121,122] may pose an important consideration in understanding resistance to PARP inhibitors. Recently published results from an in vivo ovarian cancer model showed that exposure to PARP inhibitors resulted in an increased population of CD 133^+^ CD117^+^ ovarian cancer stem cells [123]. Moreover, treatment of ovarian cancer models with PARP inhibitors in vitro resulted in no decrease in the viability of the cancer stem cell population, and this effect was demonstrated independent of BRCA status, suggesting an HR-independent mechanism of resistance [123]. The proposed ability of cancer stem cells to undergo post-treatment cell cycle arrest, as well as their superior DNA damage repair capabilities compared to non-stem cell populations [120,123,124,125], could render them particularly resistant to PARP inhibition. 

An additional mechanism of resistance is the development of the MDR1 drug efflux transporter (also known as P glycoprotein, encoded by *ABCB1*) [126]. Olaparib, rucaparib [127] and niraparib (Niraparib FDA package insert) are all P glycoprotein substrates and are subject to this mechanism of resistance. Upregulation of the MDR1 drug efflux transporter has been frequently demonstrated in chemotherapy-pretreated ovarian cancer cells [126]. *ABCB1* fusions have been proposed as a mechanism of upregulation and were found in approximately two-thirds of breast and ovarian cancer patients who had received three or more lines of paclitaxel and 26% of patients who received at least one line of liposomal doxorubicin [126]. MDR1 upregulation is likely a common mechanism of resistance, particularly in patients who have received prior chemotherapy, though assays to detect it are not part of routine practice. 

### 4.2. ATR and PARP Inhibitor Resistance 

ATR and its downstream effector CHK1 function as a cell cycle checkpoint [128] and likely play another key role in PARP inhibitor resistance. Inhibition of ATR has been shown to mitigate cell cycle arrest following chemotherapy-associated DNA damage [129]. PARP inhibitors have some cytotoxic potential, hypothesized to occur via PARP trapping leading to similar results in increased replicative stress, causing cells to exhibit a G2 cell-cycle arrest-like effect [128]. In this regard, preclinical models have clearly shown that exposure to PARP inhibition increased activation of the ATR/CHK1 pathway [130]. As expected, the pre-clinical combination of PARP inhibition and ATR inhibition led to synergistic anti-tumor activity, thought to be secondary to premature mitotic entry following unrepaired genotoxic stress [130]. ATR may play an additional role in PARP inhibitor resistance in some *BRCA1* mutant tumors. Loss of *BRCA1* typically prevents RAD51 complex binding to sites of DNA DSB [131]. Following prolonged exposure to PARP inhibitors, *BRCA1* deficient cells may regain the ability to load RAD51 to DSB through rewired pathways, dependent on ATR activity [131]. These findings further support ATR inhibition as a rational strategy to overcome PARP inhibitor resistance, particularly in tumors with loss of *BRCA1* function.

### 4.3. Mechanisms to Combat PARP Inhibitor Resistance

As we learn more about the mechanisms of PARP inhibitor resistance, new strategies are being developed to overcome them. For example, inhibitors of HSP-90, which is hypothesized to stabilize hypomorphic BRCA1, are under clinical development. A phase 1 clinical trial of the HSP-90 inhibitor onalespib in combination with olaparib is currently ongoing (NCT02898207). The potential for ATR inhibition to rescue or prevent PARP inhibitor resistance is the objective of ongoing clinical trials (NCT03462342, NCT04065269), and results are not yet available. The development of a PARP inhibitor that is not a p-glycoprotein substrate is another strategy under investigation, and a clinical trial of pamiparib, is investigating its potential benefits (NCT03933761). 

## 5. Conclusions

In conclusion, the identification of genetic perturbations in homologous recombination genes in approximately half of HGSOC has led to the development of a synthetically lethal treatment strategy using PARP inhibitors. PARP inhibitor therapy has historically been most effective for women with *BRCA* or other HR gene mutated cancers, but the ability to identify HRD or related defects in *BRCA* wild type tumors is evolving, and new diagnostic techniques may provide better predictions of treatment response. Novel treatment combinations may also expand the number of women who benefit from PARP inhibitors, and clinical trials addressing this hypothesis are ongoing. Resistance to single agent PARP inhibition inevitably develops with prolonged exposure, and several mechanisms of PARP inhibitor resistance have already been identified. Several rationally selected adjunctive therapies are hypothesized to overcome acquired resistance to PARP inhibition, and this question is the focus of many ongoing clinical trials. 

## Figures and Tables

**Table 1:** FDA-approved indications for PARP inhibitors in ovarian cancer.

PARP Inhibitor	FDA Approved Indication	Year	Important Trials	References
Olaparib	Single agent treatment for recurrent or progressive ovarian cancer with a germline *BRCA* mutation and ≥ 3 prior lines of treatment	2014	Phase 2 Proof of Concept Study, Phase 2 Study of Olaparib in Advanced Solid Tumors	[66,67]
	Maintenance therapy after response to platinum-based chemotherapy in platinum sensitive recurrent ovarian cancer irrespective of *BRCA* status	2017	Study 19, SOLO-2	[68,69,70]
	Maintenance therapy after response to first-line chemotherapy in patients with germline or somatic *BRCA* mutations	2018	SOLO-1	[71]
Rucaparib	Single agent treatment for recurrent or progressive platinum-sensitive ovarian cancer with a somatic or germline *BRCA* mutation and ≥ 2 prior lines of treatment	2016	ARIEL-2	[72]
	Maintenance therapy after response to platinum-based chemotherapy in platinum sensitive recurrent ovarian cancer irrespective of *BRCA* status	2018	ARIEL-3	[73]
Niraparib	Maintenance therapy for recurrent ovarian cancer after complete or partial response to previous platinum-based chemotherapy	2017	NOVA	[74]
	Single agent treatment for recurrent HRD-positive ovarian cancer and ≥ 3 prior lines of treatment	2019	QUADRA	[75]

**Table 2:** Ongoing phase 3 trials of olaparib monotherapy or combination therapy.

Therapy	Trial Name	Intervention	Study Population
Olaparib Alone	OrEO (NCT03106987)	Olaparib maintenance re-treatment in relapsed epithelial ovarian cancer previously treated with PARP maintenance	Recurrent epithelial ovarian, fallopian and primary peritoneal cancers with disease progression following previous PARP-maintenance with complete or partial response to subsequent treatment with platinum-based chemotherapy
Olaparib plus VEGF inhibitor	ICON-9 (NCT03278717)	Maintenance olaparib plus cediranib versus olaparib alone after treatment with platinum-based chemotherapy	Recurrent platinum-sensitive ovarian, fallopian and primary peritoneal cancers
	COCOS (NCT02502266)	Cediranib and olaparib versus cediranib or olaparib alone or standard of care chemotherapy	Recurrent platinum-resistant or refractory ovarian, fallopian and primary peritoneal cancers
Olaparib plus immunotherapy	DUO-O (NCT03737643)	Durvalumab plus platinum-based chemotherapy and bevacizumab followed by maintenance durvalumab and bevacizumab or maintenance durvalumab, bevacizumab and olaparib	Newly diagnosed advanced ovarian, fallopian and primary peritoneal cancers treated with cytoreductive surgery
	MK-7339-001/KEYLYNK-001/ENGOT-ov43 (NCT03740165)	Carboplatin / paclitaxel plus pembrolizumab and maintenance pembrolizumab and olaparib	Newly diagnosed advanced ovarian, fallopian and primary peritoneal cancers treated with cytoreductive surgery

**Table 3:** Ongoing phase 3 trials of rucaparib monotherapy or combination therapy.

Therapy	Trial Name	Intervention	Study Population
Rucaparib alone	ARIEL 4 (NCT02855944)	Rucaparib versus platinum-based chemotherapy for relapsed ovarian cancer	Recurrent advanced stage *BRCA*-mutated ovarian, fallopian and primary peritoneal cancers with ≥ 2 prior lines of chemotherapy
Rucaparib plus VEGF inhibitor	MAMOC (NCT04227522)	Rucaparib maintenance after bevacizumab maintenance following carboplatin-based first-line chemotherapy	Newly diagnosed advanced-stage ovarian, fallopian and primary peritoneal cancers, with at least stable disease after carboplatin-based chemotherapy, cytoreductive surgery and upfront + maintenance bevacizumab
Rucaparib plus immunotherapy	ATHENA (NCT03522246)	Rucaparib and nivolumab maintenance following response to primary platinum-based chemotherapy	Newly diagnosed advanced-stage ovarian, fallopian and primary peritoneal cancers treated with primary platinum-based chemotherapy and cytoreductive surgery

**Table 4:** Ongoing phase 3 trials of niraparib monotherapy or combination therapy.

Therapy	Trial Name	Intervention	Study Population
Niraparib plus immunotherapy	FIRST (NCT03602859)	Platinum-based chemotherapy versus platinum-based chemotherapy with adjuvant dostarlimab and maintenance dostarlimab and niraparib	Newly diagnosed advanced stage high-grade non-mucinous epithelial ovarian, fallopian tube or primary peritoneal cancers regardless of cytoreductive status
	ENGOT-Ov42-NSGO/AVANOVA triplet (NCT03806049)	Platinum-based chemotherapy versus niraparib-bevacizumab-dostarlimab triplet verus niraparib-bevacizumab doublet	Recurrent platinum-sensitive epithelial ovarian, fallopian tube or primary-peritoneal cancers
	ANITA (NCT03598270)	Platinum-based chemotherapy with maintenance niraparib versus platinum-based chemotherapy plus atezolizumab with maintenance niraparib and atezolizumab	Recurrent platinum-sensitive epithelial ovarian, fallopian tube or primary-peritoneal cancers with known *BRCA*-status and less than 3 lines of chemotherapy
	ROCSAN (NCT03651206)	Platinum-based chemotherapy versus niraparib monotherapy versus niraparib + dostarlimab	Metastatic or recurrent endometrial ovarian cancer or ovarian carcinosarcoma after at least 1 line of chemotherapy

## Supplementary Materials

The following are available online at https://www.mdpi.com/2072-6694/12/5/1206/s1, Table S1: Genes with full coding exonic regions in FoundationOneCDx for the detection of substitutions, insertions and deletions, and copy number alterations, Table S2: Genes with select intronic regions for the detection of gene rearrangements.

## Abbreviations

**Abbreviation**	**Definition**
H2AX	Phosphorylated histone H2A member X
ABCB1	ATP Binding Cassette Subfamily B Member 1
ALC1	Amplified in liver cancer protein 1
APLF	Aprataxin and PNKP like factor
APTX	Aprataxin
ATM	Ataxia telangiectasia mutated
ATR	Ataxia telangiectasia and Rad3 related
BARD1	BRCA associated ring domain 1
BER	Base excision repair
BID	Twice a day
BRCA1	Breast cancer 1
BRCA2	Breast cancer 2
CHK1	Checkpoint kinase 1
CSCs	Cancer stem cells
DDR	DNA damage response
DSB	double-strand break
DSD	Double-strand breaks
FDA	Food and Drug Administration
GIS	Genomic instability score
HER2	Human epidermal growth factor receptor 2
HGSOC	High grade serous ovarian cancer
HR	Homologous recombination
LOH	Loss of heterozygosity
LST	Large-scale State Transitions
MDR1	Multidrug resistance 1
MMR	Mismatch repair
MSI	Microsatellite instability
NAD	Nicotinamide adenine dinucleotide
NER	Nucleotide excision repair
NHEJ	HR and non-homologous end-joining
NuRD	Nucleosome remodeling deacetylase
PARG	Poly (ADP) ribose glycohydrolase
PARP	Poly (ADP–ribose) polymerase
PARP1	Poly (ADP–ribose) polymerase 1
PARP2	Poly (ADP–ribose) polymerase 2
PARP3	Poly (ADP–ribose) polymerase 3
PARPi	Poly (ADP–ribose) polymerase inhibitors
PFS	Progression free survival
PNKP	Polynucleotide kinase-phosphatase enzyme
RAD51	RAD51 recombinase
RAD51C	RAD51 recombinase C
RAD51D	RAD51 recombinase D
RCA	Replication Comb Assay
SEER	Surveillance, Epidemiology, and End Results
SGO	Society for Gynecologic Oncology
SigMA	signature multivariate analysis
SSB	Single-strand breaks
TAI	Telomeric Allelic Imbalance
TMB	Tumor mutation burden
XRCC1	X-ray repair cross-complementing 1
