# Supplementary material for: Exploiting the Prevalence of Homologous Recombination Deficiencies in High-Grade Serous Ovarian Cancer
Source: Cancers (Basel). 2020 May 11;12(5):1206. doi: 10.3390/cancers12051206 (PMC7281458; doi:10.3390/cancers12051206)
Supplement: Supplementary file 1 [file cancers-12-01206-s001.pdf]

Supplemental materials: Exploiting the Prevalence of Homologous Recombination Deficiencies in High-Grade Serous Ovarian Cancer

Sara Bouberhan, Lauren Philp, Sarah Hill, Linah F Al-Alem and Bo Rueda

Table S1. Genes with full coding exonic regions in FoundationOneCDx for the detection of substitutions, insertions and deletions, and copy number alterations.

(C17orf39)	BAP1	CCND3	CSF1R	ERCC4	FH	IDH1	LTK	MSH6	P2RY8	PPP2R2A	RNF43	STK11
(MLL)	BARD1	CCNE1	CSF3R	ERG	FLCN	IDH2	LYN	MST1R	PALB2	PRDM1	ROS1	SUFU
(MLL2)	BCL2	CD22	CTCF	ERRFI1	FLT1	IGF1R	MAF	MTAP	PARK2	PRKAR1A	RPTOR	SYK
ABL1	BCL2L1	CD274	CTNNA1	ESR1	FLT3	IKBKE	MAP2K1	MTOR	PARP1	PRKCI	SDHA	TBX3
ACVR1B	BCL2L2	CD70	CTNNB1	EZH2	FOXL2	IKZF1	MAP2K2	MUTYH	PARP2	PTCH1	SDHB	TEK
AKT1	BCL6	CD79A	CUL3	FAM46C	FUBP1	INPP4B	MAP2K4	MYC	PARP3	PTEN	SDHC	TET2
AKT2	BCOR	CD79B	CUL4A	FANCA	GABRA6	IRF2	MAP3K1	MYCL	PAX5	PTPN11	SDHD	TGFBR2
AKT3	BCORL1	CDC73	CXCR4	FANCC	GATA3	IRF4	MAP3K13	MYCN	PBRM1	PTPRO	SETD2	TIPARP
ALK	BRAF	CDH1	CYP17A1	FANCG	GATA4	IRS2	MAPK1	MYD88	PDCD1	QKI	SF3B1	TNFAIP3
ALOX12B	BRCA1	CDK12	DAXX	FANCL	GATA6	JAK1	MCL1	NBN	PDCD1LG2	RAC1	SGK1	TNFRSF14
AMER1	BRCA2	CDK4	DDR1	FAS	GID4	JAK2	MDM2	NF1	PDGFRA	RAD21	SMAD2	TP53
APC	BRD4	CDK6	DDR2	FBXW7	GNA11	JAK3	MDM4	NF2	PDGFRB	RAD51	SMAD4	TSC1
AR	BRIP1	CDK8	DIS3	FGF10	GNA13	JUN	MED12	NFE2L2	PDK1	RAD51B	SMARCA4	TSC2
ARAF	BTG1	CDKN1A	DNMT3A	FGF12	GNAQ	KDM5A	MEF2B	NFKBIA	PIK3C2B	RAD51C	SMARCB1	TYRO3
ARFRP1	BTG2	CDKN1B	DOT1L	FGF14	GNAS	KDM5C	MEN1	NKX2-1	PIK3C2G	RAD51D	SMO	U2AF1
ARID1A	BTK	CDKN2A	EED	FGF19	GRM3	KDM6A	MERTK	NOTCH1	PIK3CA	RAD52	SNCAIP	VEGFA
ASXL1	C11orf30	CDKN2B	EGFR	FGF23	GSK3B	KDR	MET	NOTCH2	PIK3CB	RAD54L	SOCS1	VHL
ATM	CALR	CDKN2C	EP300	FGF3	H3F3A	KEAP1	MITF	NOTCH3	PIK3R1	RAF1	SOX2	WHSC1
ATR	CARD11	CEBPA	EPHA3	FGF4	HDAC1	KEL	MKNK1	NPM1	PIM1	RARA	SOX9	WHSC1L1
ATRX	CASP8	CHEK1	EPHB1	FGF6	HGF	KIT	MLH1	NRAS	PMS2	RB1	SPEN	WT1
AURKA	CBFB	CHEK2	EPHB4	FGFR1	HNF1A	KLHL6	MPL	NT5C2	POLD1	RBM10	SPOP	XPO1
AURKB	CBL	CIC	ERBB2	FGFR2	HRAS	KMT2A	MRE11A	NTRK1	POLE	REL	SRC	XRCC2

AXIN1	CCND1	CREBBP	ERBB3	FGFR3	HSD3B1	KMT2D	MSH2	NTRK2	PPARG	RET	STAG2	ZNF217
AXL	CCND2	CRKL	ERBB4	FGFR4	ID3	KRAS	MSH3	NTRK3	PPP2R1A	RICTOR	STAT3	ZNF703

Table S2. Genes with select intronic regions for the detection of gene rearrangements.

ALK<br>introns 18, 19	BRCA1<br>introns 2, 7, 8, 12, 16, 19, 20	ETV4<br>intron 8	EZR<br>introns 9- 11	KIT<br>intron 16	MYC<br>intron 1	NUTM1<br>intron 1	RET<br>introns 7-11	SLC34A2<br>intron 4	BCL2<br>3'UTR	BRCA2<br>intron 2	ETV5<br>introns 6, 7	FGFR1<br>intron 1, 5, 17
KMT2A<br>(MLL)<br>introns 6-11	NOTCH2<br>intron 26	PDGFRA<br>introns 7, 9, 11	ROS1<br>introns 31-35	TERC<br>ncRNA	BCR<br>introns 8, 13, 14	CD74<br>introns 6-8	ETV6<br>introns 5, 6*	FGFR2<br>intron 1, 17	MSH2<br>intron 5	NTRK1<br>introns 8-11	RAF1<br>introns 4-8	RSPO2<br>intron 1
TERT<br>Promoter	BRAF<br>introns 7- 10	EGFR<br>introns 7, 15, 24-27	EWSR1<br>introns 7-13	FGFR3<br>intron 17	MYB<br>intron 14	NTRK2<br>intron 12	RARA<br>intron 2	SDC4<br>intron 2	TMPRSS2<br>introns 1-3			

© 2020 by the authors. Licensee MDPI, Basel, Switzerland. This article is an open access article distributed under the terms and conditions of the Creative Commons Attribution (CC BY) license (http://creativecommons.org/licenses/by/4.0/).
